# DNA extraction bias is more pronounced for microbial eukaryotes than for prokaryotes

**DOI:** 10.1002/mbo3.1323

**Published:** 2022-09-30

**Authors:** Anne Brauer, Mia M. Bengtsson

**Affiliations:** ^1^ Institute of Microbiology University of Greifswald Greifswald Germany; ^2^ Institute of Marine Biotechnology Greifswald Germany

**Keywords:** 18S rRNA, biofilm, DNA extraction bias, DNA preservation, microbial communities, seagrass microbiome

## Abstract

DNA extraction and preservation bias is a recurring topic in DNA sequencing‐based microbial ecology. The different methodologies can lead to distinct outcomes, which has been demonstrated especially in studies investigating prokaryotic community composition. Eukaryotic microbes are ubiquitous, diverse, and increasingly a subject of investigation in addition to bacteria and archaea. However, little is known about how the choice of DNA preservation and extraction methodology impacts perceived eukaryotic community composition. In this study, we compared the effect of two DNA preservation methods and six DNA extraction methods on the community profiles of both eukaryotes and prokaryotes in phototrophic biofilms on seagrass (*Zostera marina*) leaves from the Baltic Sea. We found that, whereas both DNA preservation and extraction method caused significant bias in perceived community composition for both eukaryotes and prokaryotes, extraction bias was more pronounced for eukaryotes than for prokaryotes. In particular, soft‐bodied and hard‐shelled eukaryotes like nematodes and diatoms, respectively, were differentially abundant depending on the extraction method. We conclude that careful consideration of DNA preservation and extraction methodology is crucial to achieving representative community profiles of eukaryotes in marine biofilms and likely all other habitats containing diverse eukaryotic microbial communities.

## Graphical abstract

DNA extraction and preservation bias is a ubiquitous issue in molecular microbial ecology that needs to be considered in every study. We compared the effect of several DNA preservation and extraction methods on the community profiles of both eukaryotes and prokaryotes in phototrophic biofilms on seagrass leaves. We found that DNA extraction bias was more pronounced for eukaryotes than for prokaryotes, which has important implications for study design when analyzing diverse multitrophic‐level microbial communities such as marine biofilms.

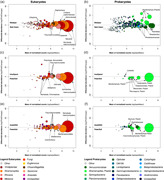

## 1 INTRODUCTION

Advances in sequencing technology and paradigm shifts in microbial ecology have led to a prolific rise in studies that use metagenomic and marker gene polymerase chain reaction (PCR) amplicon sequencing to assess microbial communities in various environments. Essential to all of these efforts is the preservation and extraction of DNA from environmentally or host‐associated microbial communities. It is well known that the choice of DNA preservation and extraction method can impact the perceived relative abundance of microbial taxa in microbial communities (e.g., Martin‐Laurent et al., 2001). Differences in community composition depending on the DNA extraction method are referred to as extraction bias, which can have various causes, many of which are linked to the ability to lyse microbial cells (Koid et al., 2012). A wide variety of commercial kits and custom protocols have been developed to provide representative and reproducible DNA extraction from different sample types. For some environments, extraction bias has been evaluated by comparing the outcome of different extraction protocols, in some cases, leading to general recommendations on method choice (e.g., Albertsen et al., 2015; Weber et al., 2017). A majority of existing studies have focused on prokaryotic communities, reflecting an emphasis on bacteria and archaea in molecular microbial ecology.

However, in most natural environments, microbial eukaryotes are abundant and diverse and play essential roles in ecosystem processes. Whereas they have traditionally been studied using microscopic methods, studies using molecular methods have revealed novel taxa that escape microscopic detection or identification (Jones et al., 2011; Liu et al., 2009). In the wake of numerous influential studies on prokaryote diversity in various ecosystems, microbial eukaryotes are receiving renewed attention by taking advantage of available high‐throughput sequencing technologies (Delmont et al., 2022; Lima‐Mendez et al., 2015).

Due to a high diversity of cell envelopes found in microbial eukaryotes, ranging from single membranes in ameboid protists to silica frustules of diatoms or thick cellulose cell walls of green algae, effective cell lysis and subsequent DNA recovery pose unique challenges. Despite this, extraction bias has so far received little attention in surveys of microbial eukaryotes (but see Donn et al., 2008; Koid et al., 2012; Mäki et al., 2017; Santos et al., 2015; Vesty et al., 2017). In addition, microbial eukaryotes and prokaryotes are intermingled in most microbial communities, and extraction methods that recover DNA well from a variety of eukaryotes and prokaryotes are needed to achieve an accurate representation of microbial community composition.

Here, we compared the effect of different popular commercial and custom DNA extraction methods on the perceived community composition of prokaryotes and eukaryotes in marine phototrophic biofilms growing on seagrass leaves. We aimed to assess whether extraction bias affects microbial eukaryotes and prokaryotes at a similar magnitude in the same environment and whether this bias depends on the sample preservation method.

Phototrophic biofilms are known to be complex microbial ecosystems including members of all three domains of life, encompassing several trophic levels (Bengtsson et al., 2018). This is a property that they share with many other microbial habitats, including soils, sediments, and plankton. Biofilm material from the leaves of the seagrass *Zostera marina* was rubbed off with a cotton swab. We used two different methods to preserve the DNA in the biofilms before extraction: biofilms were either suspended in sterile seawater, pelleted by centrifugation, frozen in liquid N_2_, and stored at −20°C (hereafter referred to as flash frozen) or they were suspended in RNAlater, pelleted, and stored at +4°C (hereafter referred to as RNAlater). To ensure comparable results, the different extraction methods started with pellets (in triplicate) of similar masses from the same suspension (one flash frozen suspension and one RNAlater suspension). The six different extraction methods that were tested (summarized and detailed in Table A1) varied in lysis method (five mechanical vs. 1 enzymatic), lysing matrix, and intended sample material (soil, biofilm, and general). We used Illumina MiSeq sequencing of amplicons of small subunit ribosomal RNA (SSU rRNA) gene fragments of prokaryotes (16S rRNA) and eukaryotes (18S rRNA) to assess the microbial community composition of the biofilms (see the Appendix A for detailed descriptions of extraction methods and sequencing).

## 2 RESULTS AND DISCUSSION

### 2.1 Extraction bias was more pronounced for eukaryotes than for prokaryotes

**Figure 1:**
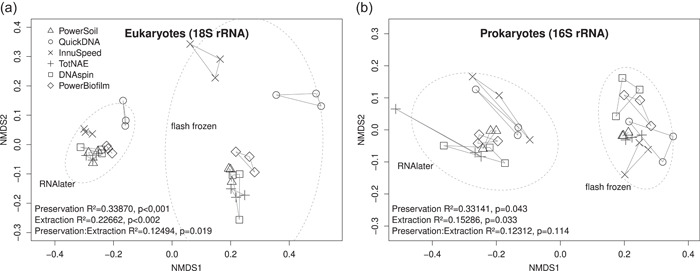
Comparison of communities of epibiotic microbial eukaryotes (a) and prokaryotes (b) on *Zostera marina* treated with different DNA preservation methods and DNA extraction methods. The six different extraction methods (different shapes) that were tested are summarized and detailed in Table A1. nMDS ordinations based on Bray–Curtis distances were calculated from Hellinger transformed sequence variant counts; dashed lines indicate the 95% confidence interval of the factor preservation method. PERMANOVA results are indicated in the lower left corners, *R* ^2^ × 100 corresponds to the % of variation explained. nMDS, nonmetric multidimensional scaling; PERMANOVA, permutational multivariate analysis of variance; and rRNA, ribosomal RNA.

**Figure 2:**
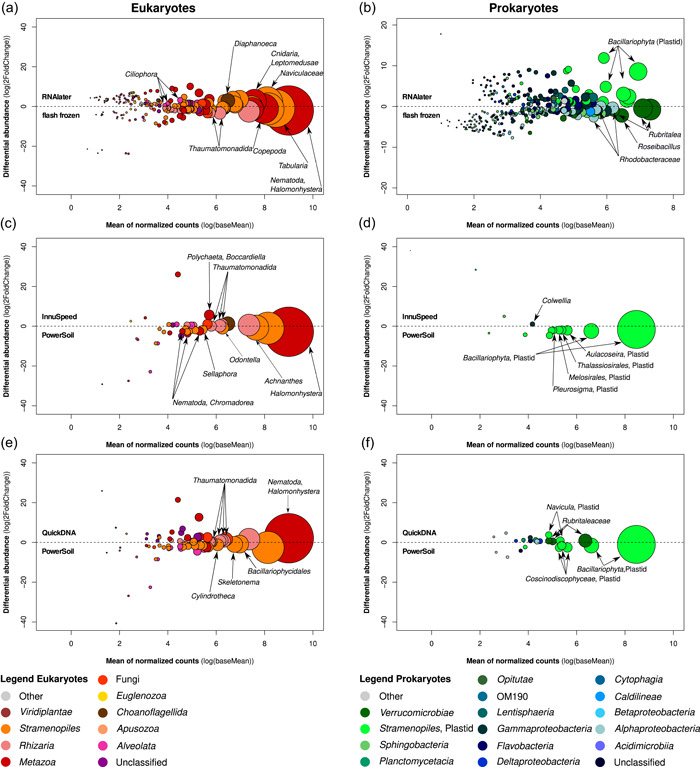
Significantly differentially abundant taxa (ASVs, *p* < 0.01 are shown) in the epibiotic microbial eukaryotic (a, c, e) and prokaryotic (b, d, f) communities on *Zostera marina* treated with the two different preservation (a, b) or selected DNA extraction methods (c–f) as detected by DeSeq2 parametric Wald test. Point diameter is scaled by the abundance of the ASVs. (c, d) Communities extracted using the InnuSpeed method compared to the PowerSoil method. (e, f) Communities extracted using the QuickDNA method compared to the PowerSoil method. Taxa names on arrows indicate the finest taxonomic resolution for selected ASVs. Pairwise comparisons with more than 10 significant differentially abundant taxa are shown here; see Figure A5 for the remaining comparisons

The extraction method explained a significant amount of variation (permutational multivariate analysis of variance [PERMANOVA] *p* < 0.05) in both eukaryotes and prokaryotes, confirming the presence of extraction bias for both groups (Figure 1). However, extraction bias was more pronounced for eukaryotes (22.7% of variation explained, *p* < 0.01) than for prokaryotes (15.3% of variation explained, *p* < 0.05). Two of the tested extraction methods, the InnuSpeed method using the InnuSpeed Soil DNA kit (Analytik Jena) and the QuickDNA method using the QuickDNA Universal kit (Zymo Research), gave rise to more distinct eukaryote community compositions compared to the other four methods, especially for flash frozen samples (Figure 1a). These two methods were characterized by more gentle lysis conditions, weak bead beating (smaller beads than in the other tested methods; see Table A1) and enzymatic lysis, compared to the other methods that use harsh bead beating, indicating that incomplete lysis of some eukaryotic cells may underlie the observed pattern. However, when investigating which eukaryotic taxa were differentially abundant in these methods, we found that metazoans, especially nematodes and annelids, and rhizarian (Cercozoa) amplicon sequence variants (ASVs) were overrepresented in samples from the QuickDNA method compared to the PowerSoil method using the PowerSoil DNA isolation kit (Figure 2e), a representative example of the methods based on mechanical lysis. Nematodes and annelids are generally soft‐bodied, and, therefore, do not require harsh mechanical lysis for DNA recovery. Hence, their overrepresentation in the QuickDNA method may in part reflect a higher recovery of PCR‐amplifiable nematode DNA, perhaps due to selective fragmentation of nematode DNA in the other, mechanical lysis‐based, methods. In contrast, several diatom sequence variants were underrepresented in samples extracted using the QuickDNA method (Figure 2e), indicating that enzymatic lysis might inefficiently lyse their silica frustules. This result was also supported by an underrepresentation of diatom plastid sequence variants (16S rRNA; Figure 2f) in the samples extracted using the QuickDNA method, while Rubritaleaceae ASVs (*Verrucomicrobia*) were overrepresented. Using the InnuSpeed kit, Polychaeta (Metazoa) and Cercozoa (*Rhizaria*) ASVs were overrepresented, while diatom ASVs and some nematode (Metazoa) ASVs were underrepresented (Figure 2c). For example, an ASV was classified as *Halomonhystera disjuncta* (nematode), which was overrepresented in the QuickDNA method. Several diatom plastid sequences were underrepresented with the InnuSpeed kit, indicating that the weak bead beating was not sufficient to completely lyse the silica frustules (Figure 2d).

### 2.2 Preservation protocol had a stronger influence on community composition than the extraction method

Preservation protocol was the strongest explanatory variable for both prokaryotic (33.1% of variation explained, *p* < 0.05) and eukaryotic communities (33.9% of variation explained, *p* < 0.01), illustrated by a clear separate clustering of RNAlater and flash‐frozen samples in the nonmetric multidimensional scaling (nMDS) ordinations (Figure 1). Preservation bias affected mainly Diatoms, Alveolata, *Cnidaria*, and *Bacillariophyta* (plastids), which were overrepresented in the RNAlater‐preserved samples, while Nematodes, Cercozoa, and *Rubritaleaceae* (*Verrucomicrobia*) were underrepresented (Figure 2a,b). A possible cause could be the different guanine–cytosine content (GC) contents of DNA in the different organisms, as Gray et al. (2013) showed that bacteria with a high GC content are poorly recovered from samples conserved with RNAlater. Another factor could be the Gram status of prokaryotes (Watson et al., 2019). However, the overall community composition patterns remained comparable (Figure A4), and no major groups of organisms were excluded from the data set in either preservation protocol treatment. This is consistent with recent findings of Burgunter‐Delamare et al. (2022). Interestingly, RNAlater‐treated samples appeared to be less impacted by DNA extraction bias in the case of eukaryotic communities, illustrated by the smaller 95% confidence interval in Figure 1a. This may suggest that RNAlater affects the structural integrity of cells, making them easier to lyse and thereby rendering the effect of mechanical versus enzymatic lysis less decisive.

The optimal preservation protocol for a given study depends on different factors like practicability under field conditions, perishability of the sample material, and expected storage time of the samples. It might, for example, not be possible to transport liquid nitrogen to remote sampling sites, on long field trips, or in small boats (Burgunter‐Delamare et al., 2022). In this case, prefilled tubes with RNAlater would be better suited. Another consideration is how prone the sample material is to changes during handling. Flash freezing in liquid nitrogen sometimes requires preprocessing of the samples such as filtration or other means of sample concentration as well as packaging in cryovials. This handling could lead to the degradation of nucleic acids. Similarly, degradation can take place upon thawing of flash‐frozen samples, as some handling before DNA extraction is typically difficult to avoid. Storage time is another critical aspect influencing the preservation method choice. Frozen samples can stay stable for years, while storage in RNAlater requires faster processing (e.g., DNA extraction within weeks after sampling).

### 2.3 DNA yield does not impact community composition

**Figure A1:**
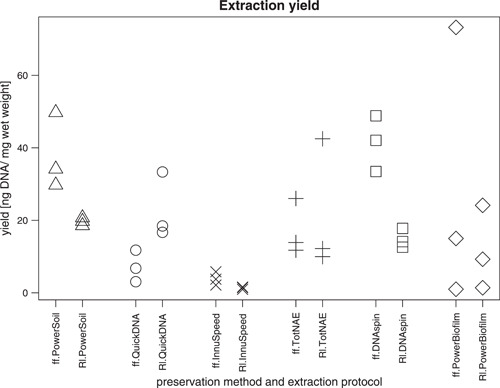
DNA yield across the different DNA preservation and extraction methods

**Figure A4:**
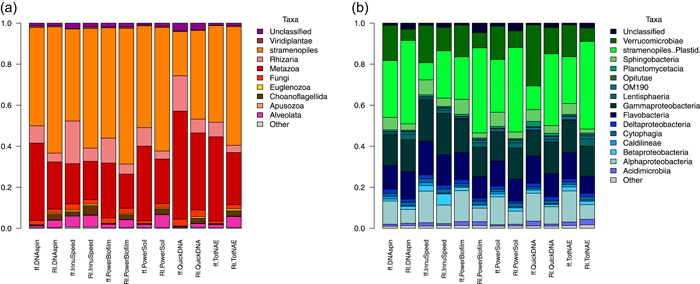
Relative abundances of the epiphytic microbial eukaryotic (a) and prokaryotic (b) taxa in the biofilm covering *Zostera marina* leaves

The DNA yield differed significantly among extraction methods (Kruskal–Wallis rank‐sum test, *p* < 0.05), with the highest DNA yields observed for the PowerSoil and DNASpin kits in the flash‐frozen samples (Figure A1). The QuickDNA kit was the only one that resulted in a higher yield on RNAlater‐preserved samples than on flash‐frozen samples. DNA yield did not significantly explain the variation in perceived community composition across prokaryotic and eukaryotic samples (PERMANOVA, *p* > 0.2 and *p* > 0.05, respectively), indicating that factors that affect the overall yield are different from those giving rise to DNA extraction bias. This is reassuring since extraction yield can vary substantially even between replicate samples under the same extraction method (see e.g., PowerBiofilm method, Figure A1), but this does not compromise the reproducibility of community composition patterns (Vishnivetskaya et al., 2014).

## 3 CONCLUSIONS

Most microbial DNA extraction methods have been developed and optimized for prokaryotes and may therefore be inadequate for microbial eukaryotes, which have a high diversity of cell envelopes, posing unique challenges for effective cell lysis and subsequent DNA recovery. It is unlikely that we will ever arrive at one optimal methodology that captures all organism groups without bias. It is also not the aim of this study to offer specific recommendations for DNA preservation or extraction methods or kits. Commercial buffers and kits such as those used in this study can be discontinued or the recipe can change (this was recently the case with the PowerSoil kit, which was discontinued as MoBio was taken over by Qiagen), thereby making specific recommendations meaningless within a short time. However, in light of our results, we recommend that the extraction and preservation method should be chosen carefully depending on the specific groups of interest in the focal ecosystem. If soft‐bodied microbes like nematodes and other microscopic metazoans are especially important to recover, gentle lysis methods such as chemical and enzymatic lysis may be preferred over harsh mechanical lysis. Conversely, lysis of organisms with hard cell walls or frustules, such as diatoms, may benefit from mechanical methods such as bead beating. Finally, although preservation via RNAlater does impact perceived community composition in both eukaryotes and prokaryotes significantly, it still offers representative community profiles and even appears to mitigate the effect of DNA extraction bias for eukaryotes. Therefore, we recommend preservation in RNAlater (and other similar buffers) as a practical and adequate alternative to flash‐freezing.

## AUTHOR CONTRIBUTIONS

**Anne Brauer**: Conceptualization (supporting), data curation (equal), formal analysis (lead), investigation (lead), methodology (equal), validation (equal), and visualization (lead) of the study, writing of the manuscript – original draft (equal) and writing of the manuscript – review and editing (equal). **Mia M. Bengtsson**: Conceptualization (lead), data curation (equal), formal analysis (supporting), funding acquisition (lead), investigation (supporting), methodology (equal), project administration (lead), resource procurement (lead), supervision (lead), validation (equal), and visualization (supporting) of the study, writing of the manuscript – original draft (equal) and writing of the manuscript – review and editing (equal).

## CONFLICT OF INTEREST

None declared.

## ETHICS STATEMENT

None required.

## Figures and Tables

**Figure A2:**
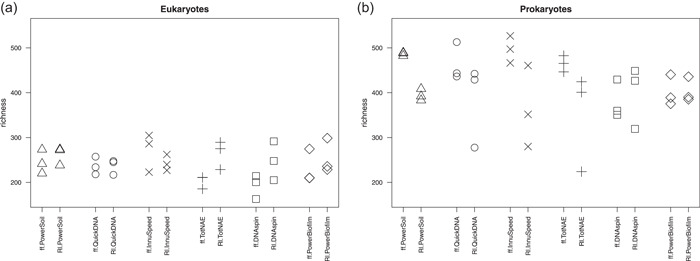
Rarefied richness of microbial (a) eukaryotes and (b) prokaryotes across the different DNA preservation and extraction methods.

**Figure A3:**
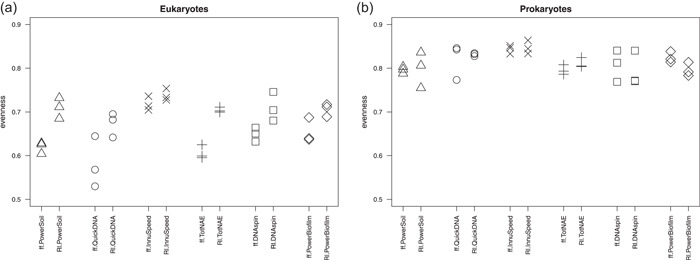
Evenness of microbial (a) eukaryotes and (b) prokaryotes across the different DNA preservation and extraction methods.

**Figure A5:**
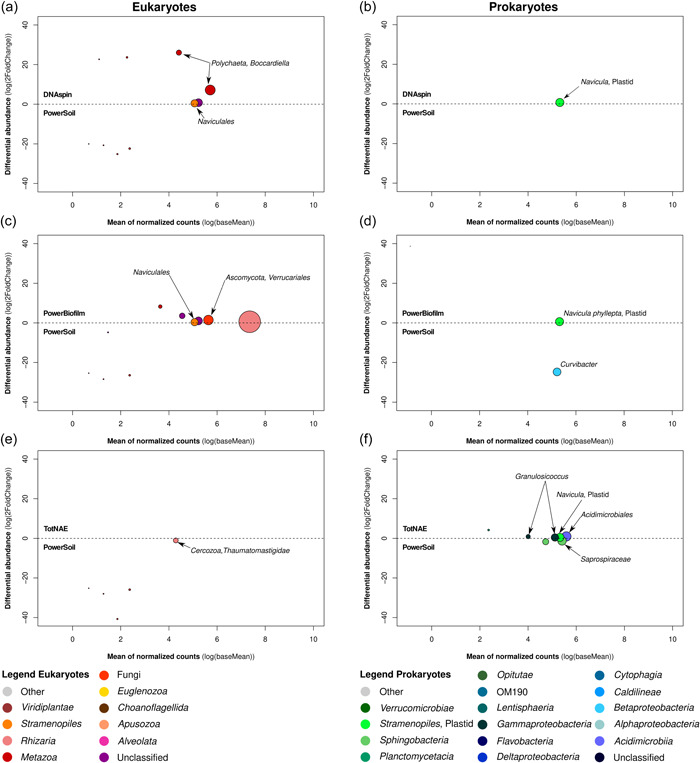
Significantly differentially abundant taxa (ASVs, *p* < 0.01 are shown) in the epibiotic microbial eukaryotic (a, c, e) and prokaryotic (b, d, f) communities on *Zostera marina* treated with different DNA extraction methods as detected by the DeSeq2 parametric Wald test. Point diameter is scaled by the abundance of the ASVs. (a, b) communities extracted by the DNAspin method compared to the PowerSoil method. (c, d) Communities extracted by the PowerBiofilm method compared to the PowerSoil method. (e, f) Communities extracted by the TotNAE method compared to the PowerSoil method. Taxa names on arrows indicate the finest taxonomic resolution for selected ASVs.

## Data Availability

The data sets generated and analyzed during the current study are available at https://doi.org/10.13140/RG.2.2.28409.54888. Sequences generated during the current study are available in the NCBI short read archive under the project number PRJNA389390 and accession numbers SRX29110 92 and 93, SRX29111 20–29, 50–59, 76–79, and 90–99 for eukaryotes, and SRX29110 98 and 99, SRX29111 00–19, 40–49, and 60–69 for prokaryotes: https://www.ncbi.nlm.nih.gov/bioproject/PRJNA389390.

## APPENDIX A

### A.1. MATERIAL AND METHODS

**A.1.1. Sample collection**

Seagrass plants were collected by diving from a depth of approximately 3 m in the Kieler Förde near Falkenstein (54°24′9″ N, 10°11′46″ E) in the summer of 2015. They were planted in pairs in plastic boxes in a tank. Samples for the experiment were collected on January 31, 2016. For this purpose, the plastic boxes were removed from the tank and 16 single plants with rhizomes were picked and the roots were rinsed with water from the tank to get rid of sediments. Then, each plant was put in a plastic bag with a little water from the tank in it and kept cool (0°C to +4°C) until sample processing the following day.

**A.1.2. Sample preparation and preservation**

To relate to the total leaf surface, the leaf widths and lengths of each leaf of all the plants were measured. Then, the leaves were rinsed with sterile filtered seawater (pore size = 0.2 µm) and the biofilm attached to the leaves was rubbed off with a sterile cotton swab. For the flash‐frozen samples, the biofilm material was suspended in sterile filtered seawater, aliquoted to 1.5 ml reaction tubes, centrifuged to pellets, frozen in liquid N_2_, and stored at *−*20°C. To preserve the DNA in RNAlater, the biofilm material was suspended in RNAlater, aliquoted to 1.5 ml reaction tubes, centrifuged into a pellet, and stored at 4°C until DNA extraction.

**A.1.3. DNA extraction**

A.1.3.1. DNA extraction protocol comparison

We tested six different DNA extraction protocols (Table A1) in triplicate for the flash‐frozen and the RNAlater‐preserved biofilm samples. Before the extraction, the wet weight of each pellet was determined. For this purpose, the samples were centrifuged and all the remaining liquid was removed. The samples preserved in RNAlater were suspended in 1400 µl of phosphate‐buffered saline (pH = 7.4, *T* = 4°C) and pelleted before the extraction to wash out the RNAlater that could interfere with the extraction buffers. The pellets (thawed flash‐frozen and washed with RNAlater) were then suspended in the first extraction buffer of the respective kit and transferred to the respective reaction tubes to start DNA extraction. The extractions were performed according to the manufacturer's instructions, with slight modifications. A short overview of each extraction protocol and the conducted changes are described below. Centrifugation was performed at 15,000 *g* and room temperature (RT) unless otherwise specified.

(a) *PowerSoil®DNA isolation kit* (Mo Bio Laboratories, hereafter referred to as PowerSoil)
  Cells were lysed by bead beating in the provided PowerBead tubes using a FastPrep®‐24 homogenizer (MP Biomedical) at 5 m s^*−*1^ for 45 s in the presence of sodium dodecyl sulfate. Subsequent centrifugation was extended to 45 s. Non‐DNA material was precipitated by incubation with two different Inhibitor Removal Technology®solutions at 4°C for 5 min. The DNA solution was then mixed with a highly concentrated salt solution and applied onto a SpinFilter with a silica membrane. Afterward, the DNA was washed with an ethanol‐based wash solution and was eluted with 100 µl of elution buffer.
(b) *Quick‐DNA™Universal kit* (Zymo Research, hereafter referred to as QuickDNA)
  The samples were incubated in the presence of a buffer and proteinase K in a water bath at 55°C for 3 h and were vortexed every 30 min. After centrifugation for 1 min, 220 µl of the supernatant was mixed with 440 µl of genomic binding buffer and applied to a spin column with a silica membrane. The DNA was washed three times with the two different solutions before elution with 100 µl of elution buffer.
(c) *innuSPEED Soil DNA kit* (Analytik Jena, hereafter referred to as innuSPEED)
  Samples were first incubated with a lysis solution in lysis tube B at 95°C for 20 min and were vortexed every 5 min. Bead beating was performed in a FastPrep®‐24 homogenizer at 5 m s^*−*1^ for 45 s. Subsequently, the samples were centrifuged for 5 min and 300 µl of the supernatant was mixed with 300 µl of binding solution and applied to a spin filter with a silica membrane. The samples were washed three times with two different solutions before incubation for 2 min at RT with 100 µl of elution buffer and the first elution. The extract was then mixed with the washing solution and binding solution, applied to a clean spin filter, and washed again. After incubation for 3 min at RT with 80 µl of elution buffer, the final DNA extracts were eluted.
(d) *Total nucleic acid extraction protocol* (described by Griffiths et al. (2000) modified by Urich et al. (2008), followed by RNAse treatment and cleaning with DNA Clean & Concentrator™‐5 (Zymo Research Europe), hereafter referred to as TotNAE).
  Centrifugation was performed at 17,000 *g* and 4°C. First, the samples were added together with 500 µl of extraction buffer (5% cetyltrimethylammonium bromide, 120 mmol KaPO_4_, pH = 8) and 500 µl of phenol–chloroform–isoamyl alcohol (25:24:1) to lysis tubes. Bead beating was performed using a FastPrep®‐24 homogenizer at 5 m s^*−*1^ for 45 s. The samples were centrifuged for 10 min. The aqueous phase was mixed by inverting with chloroform–isoamyl alcohol (24:18) and centrifuged for 5 min. To the aqueous phase, 1 µl of glycogen and 1000 µl of polyethylene glycol‐6000 were added. Samples were left for precipitation on ice for 2 h. The nucleic acids were pelleted by centrifugation for 60 min. The supernatant was discarded and 1000 µl of ice‐cold ethanol (70%) was added. After centrifugation for 10 min, the pellet was dried and then dissolved in 100 µl of PCR water. The DNA extracts were aliquoted in 50 µl portions and a 0.5 µl RNAse inhibitor was added to one of them. To another, RNAse A was added to a final concentration of 25 µg ml^*−*1^ and the samples were incubated for 20 min at RT. Then, two volumes of DNA binding buffer were added to the samples and they were applied to a spin column with a silica membrane. The DNA was washed twice and then eluted in 40 µl of elution buffer.
(e) *FastDNA™SPIN kit for soil* (MP Biomedical, hereafter referred to as DNAspin)
  The samples were lysed in sodium phosphate buffer using a FastPrep®‐24 homogenizer at 6 m s^*−*1^ for 40 s. After centrifugation for 10 min, 800 µl of the supernatant was mixed by gentle shaking with a protein precipitation solution and incubated at RT for 10 min. After centrifugation for 5 min, an equal volume of the silica‐based binding matrix was added to 800 µl of the supernatant. The samples were shaken by hand for 5 min. This mixture was added to a SPIN filter and washed once. After the addition of 100 µl of elution buffer, the samples were incubated at 55°C for 5 min and then centrifuged for 2 min to elute the DNA.
(f) *PowerBiofilm®DNA isolation kit* (Mo Bio Laboratories, hereafter referred to as PowerBiofilm)

**Table A1:** Overview of the compared DNA extraction methods

Method	Manufacturer	Lysis	Lysing matrix	Intended sample material
(a) PowerSoil	MoBio Laboratories	m	Garnet 0.7 mm	Soil
(b) QuickDNA	Zymo Research	e	Proteinase K	Liquid biological samples
(c) innuSPEED	Analytik Jena	m	Beads 0.09–0.15 mm	Soil
(d) TotNAE	Noncommercial	m	Garnet and bead 6 mm	Optimized for soil
(e) DNAspin	MP Biomedical	m	Beads 0.1–4 mm	Soil
(f) PowerBiofilm	MoBio Laboratories	m	Beads 0.1–2.4 mm	Biofilm

Abbreviations: e, enzymatical; m, mechanical.

The first buffer was heated to 55°C before use. Samples were incubated at 65°C for 5 min and then lysed by bead beating using a FastPrep®‐24 homogenizer at 5 m s^*−*1^ for 45 s. After centrifugation for 2 min, 400 µl of the supernatant was mixed with an Inhibitor Removal Technology® solution and incubated at 4°C for 7 min. The precipitate was removed and the supernatant was mixed with a highly concentrated salt solution (for samples M17 and M18, the highly concentrated salt solution of the PowerSoil®DNA kit was used, as there was not enough left). Samples were applied onto a SpinFilter with a silica membrane, washed twice with an ethanol‐based wash solution, and eluted with 100 µl of elution buffer. Table A1 overview of the compared DNA extraction methodsMethod Manufacturer Lysis Lysing matrix Intended sample material(a) PowerSoil MoBio Laboratories m Garnet 0.7 mm Soil(b) QuickDNA Zymo Research e Proteinase K Liquid biological samples(c) innuSPEED Analytik Jena m Beads 0.09–0.15 mm Soil(d) TotNAE Noncommercial m Garnet and bead 6 mm Optimized for soil(e) DNAspin MP Biomedical m Beads 0.1–4 mm Soil(f) PowerBiofilm MoBio Laboratories m Beads 0.1–2.4 mm BiofilmAbbreviations: e, enzymatical; m, mechanical.

**A.1.4. DNA quality and quantity**

DNA concentration was measured by fluorescence spectroscopy with a Qubit®3.0 fluorometer (Thermo Fisher Scientific) using the Qubit®dsDNA HS Assay kit. The assay was performed according to the manufacturer's instructions. For each sample, 195 µl of the working solution was prepared, mixed in an assay tube with 5 µl of DNA extract, and incubated for 2 min at RT before measurement. The integrity of the extracted DNA was analyzed by gel electrophoresis with a 1% agarose gel stained with ethidium bromide.

**A.1.5. Illumina amplicon sequencing**

Paired‐end amplicon sequencing with a read length of 300 bp was performed by LGC Genomics on an Illumina MiSeq V3 (Illumina) platform (PCR amplification, Illumina MiSeq library preparation, and sequencing (V3 chemistry)). To access the prokaryotic community, the V4 region of the 16S rRNA gene with primers (515F: 5′‐GTG YCA GCM GCC GCG GTA A‐3′ and 806R: 5′‐GGA CTA CNV GGG TWT CTA AT‐3′) spanning the V4 hypervariable region of 16S rDNA was used (Walters et al., 2016). Eukaryotes were investigated using (F‐1183mod: 5′‐AAT TTG ACT CAA CRC GGG‐3′ and R‐1443mod: 5′‐GRG CAT CAC AGA CCT G‐3′) primers targeting the V7 region of the 18S rDNA (Ray et al., 2016). Primers were coupled to custom adaptor‐barcode constructs. Sequences are available in the NCBI short read archive under the project number PRJNA389390 and accession numbers SRX29110 92 and 93, SRX29111 20–29, 50–59, 76–79, and 90–99 for eukaryotes and SRX29110 98 and 99, SRX29111 00–19, 40–49, and 60–69 for prokaryotes.

**A.1.6. Sequence processing**

Clipped sequences (adaptor and primer sequence remains removed) were processed using the DADA2 package (Callahan et al., 2016) in R (version 1.2.0) (R Core Team, 2018). Briefly, sequences were truncated to 200 bp length, filtered (maxEE = 2, truncQ = 2), dereplicated, and error rates were estimated with the maximum possible error estimates from the data as the initial guess. Sample sequences were inferred and paired reads were merged. To remove chimeric sequences the removeBimeraDenovo function was used. The resulting unique sequence variants (ASVs) were taxonomically classified using the lowest common ancestor approach implemented in CREST (Lanzen et al., 2012) based on the Silva database (Pruesse et al., 2007).

**A.1.7. Statistical analysis**

Statistical analysis was carried out in R (R Core Team, 2018) using functions from the vegan package (Oksanen, 2022) and the deseq.2 package (Love et al., 2014). Similarities in ASV composition were visualized using nonmetric multidimensional scaling (vegan function metaMDS) of Bray–Curtis distances calculated from Hellinger transformed ASV counts. To assess the influence of preservation and extraction methods on community composition, a PERMANOVA test (vegan function adonis) was performed. Differentially abundant ASVs were calculated using a parametric Wald test in deseq.2. Pairwise differential abundances were extracted for the RNAlater versus the flash‐frozen samples and for the PowerSoil method versus all other tested DNA extraction methods. Only differentially abundant taxa *p* < 0.01 were considered.

Figure A2, A3
